# Local Left Ventricular Epicardial J Waves and Late Potentials in Brugada Syndrome Patients with Inferolateral Early Repolarization Pattern

**DOI:** 10.3389/fphys.2017.00014

**Published:** 2017-01-26

**Authors:** Satoshi Nagase, Masamichi Tanaka, Hiroshi Morita, Koji Nakagawa, Tadashi Wada, Masato Murakami, Nobuhiro Nishii, Kazufumi Nakamura, Hiroshi Ito, Tohru Ohe, Kengo F. Kusano

**Affiliations:** ^1^Department of Cardiovascular Medicine, National Cerebral and Cardiovascular CenterOsaka, Japan; ^2^Departments of Cardiovascular Medicine, Okayama University Graduate School of Medicine, Dentistry, and Pharmaceutical SciencesOkayama, Japan; ^3^Departments of Cardiovascular Therapeutics, Okayama University Graduate School of Medicine, Dentistry, and Pharmaceutical SciencesOkayama, Japan; ^4^Department of Cardiovascular Medicine, Sakakibara Heart Institute of OkayamaOkayama, Japan

**Keywords:** Brugada syndrome, J wave syndrome, early repolarization, delayed potential, left ventricle, epicardium

## Abstract

**Background:** Brugada syndrome (BrS) is characterized by J-point or ST-segment elevation on electrocardiograms (ECGs) and increased risk of ventricular fibrillation (VF). In BrS, epicardial depolarization abnormality with delayed potential on the right ventricular outflow tract is reportedly the predominant mechanism underlying VF. Yet VF occurrence is also associated with early repolarization (ER) pattern in the inferolateral ECG leads, which may represent the inferior and/or left lateral ventricular myocardium. The aim of this study was to examine epicardial electrograms recorded directly at the left ventricle (LV) in BrS patients after VF episodes.

**Methods:** In 12 BrS patients who had experienced VF episodes and 17 control subjects, a multipolar catheter was introduced into the left lateral coronary vein for unipolar and bipolar electrogram recordings at the LV epicardium. Both inferior and lateral ER patterns on ECG were observed in three BrS patients and six control subjects.

**Results:** In the epicardium, prominent J waves were detected using unipolar recording, and potentials after the QRS complex were detected using bipolar recording in three of the 12 BrS patients. These three patients also showed both inferior and lateral ER patterns on ECG. Neither prominent J waves nor potentials after the QRS complex were recorded at the endocardium of the LV in any of these three patients; nor were they seen at the epicardium in any of the control subjects. These features were accentuated on pilsicainide administration (*n* = 2) but diminished on constant atrial pacing (*n* = 3) and isoproterenol administration (*n* = 1). The J waves observed through unipolar recording coincided with the potentials after QRS complex observed through bipolar recording and with the inferolateral ER patterns on ECG.

**Conclusions:** We recorded prominent J waves in unipolar electrogram and potentials after QRS complex in bipolar electrogram at the LV epicardium in BrS patients with global ER pattern. The prominent J waves coincided with the potentials after QRS complex and the inferolateral ER pattern on ECG. The characteristics of the inferolateral ER pattern on ECG in these patients primarily represent depolarization feature.

## Introduction

Brugada syndrome (BrS) and early repolarization syndrome (ERS) are fatal arrhythmic diseases characterized by unique electrocardiographic patterns involving the J wave or ST segments and an increased risk of sudden cardiac death due to polymorphic ventricular tachycardia (PVT) or ventricular fibrillation (VF) (Brugada and Brugada, 1992; Haissaguerre et al., 2008). Recently, Antzelevitch et al. proposed that J wave syndrome (JWS)—characterized by early repolarization (ER) pattern on electrocardiogram (ECG) and an increased risk of PVT/VF—is an integrative classification (Antzelevitch and Yan, 2010; Antzelevitch et al., 2016). They reported that BrS and ERS have similar mechanisms and that BrS can overlap with ERS. Several other studies have also reported that the presence of inferolateral ER is associated with fatal arrhythmic events in patients with BrS (Kamakura et al., 2009, 2013; Sarkozy et al., 2009; Aizawa et al., 2012; Kaneko et al., 2014; Tokioka et al., 2014). Accordingly, analysis of inferolateral ER patterns is clinically and electrophysiologically important in patients with BrS. In general, ECG is obtained through unipolar or semi-unipolar recording with two electrodes placed far apart. Consequently, the ER patterns analyzed in electrophysiological studies (EPS) are often acquired through unipolar recording.

Experimental studies have suggested that one hypothetical mechanism of the ER pattern in JWS is a prominent transient outward potassium current (Ito)-mediated action potential notch in epicardial cells, which creates a transmural voltage gradient (Antzelevitch and Yan, 2010). Another hypothesis identifies the mechanism of ST-segment elevation as excitation failure caused by current-to-load mismatch (Hoogendijk et al., 2011; Ten Sande et al., 2015). According to the current to load mismatch hypothesis in increase of Ito (for example by a lowering heart rate) will decrease the net inward current and will promote conduction failure/slowing, whereas a decrease of Ito (for example by administration of quinidine) will counteract conduction failure/slowing. In BrS, we have identified a prominent potential after the QRS complex in the epicardium but not in the endocardium at the right ventricular outflow tract (RVOT) (Nagase et al., 2002). Several previous papers have also reported delayed and fractionated potentials in BrS in bipolar recordings in the RVOT epicardium (Coronel et al., 2005; Nademanee et al., 2011; Ten Sande et al., 2015). Given this, depolarization abnormalities on the RVOT epicardium must constitute an important mechanism for VF in BrS. The left ventricular (LV) epicardium, on the other hand, has not been thoroughly examined for similar findings in BrS in either unipolar or bipolar recordings.

We recently published a case report detailing a LV epicardial electrogram with unipolar and bipolar recordings in a patient with ERS (Nakagawa et al., 2014). This case exhibited prominent J waves and potentials after the QRS complex on the epicardium of the lateral LV, but not within the endocardium on the opposite side. These findings were accentuated by pilsicainide administration and suppressed by constant atrial pacing and isoproterenol administration. Moreover, the epicardial J waves coincided with the J-waves on ECG. Although administration of pilsicainide exacerbates epicardial J waves, ER did not conform to this tendency on ECG. This might be due to a transmural or far-field conduction delay causing the J wave to merge with the S wave (Antzelevitch et al., 2016; Meijborg et al., 2016). Either way, however, epicardial unipolar recording could be important for the understanding of ER or ST-segment patterns on ECG, especially in JWS patients.

To date, no clinical study has recorded and examined LV epicardial electrograms in patients with BrS. In the present study, we directly recorded epicardial electrograms in the LV and examined the characteristics of the LV epicardial potential in patients with BrS and control subjects.

## Materials and methods

### Study population

Twelve patients with BrS (mean age 43 ± 11 years, 100% male, Table 1) and 17 control subjects (mean age 48 ± 19 years, 76% male, Table 2) were evaluated in this study. Age and gender were not significantly different between the two groups. All patients with BrS had experienced more than one episode of VF. BrS was defined as the manifestation of type 1 ECG, which is characterized by a coved-type ST segment elevation ≥0.2 mV followed by a negative T wave in leads V1 or V2 at the second, third or fourth intercostal space in the presence or absence of a class IC antiarrhythmic drug (pilsicainide) (Priori et al., 2013). Routine examinations, including cardiac echocardiography, right and left ventriculography, and coronary angiography, did not show any evidence of structural heart disease in any of the BrS patients. Cardiac echocardiography did not show any evidence of structural heart disease or Brugada-type ECG in the control subjects. Pilsicainide challenge was performed in 16 out of 17 control subjects and was negative in all 16. The characteristics of BrS patients are shown in Table 1. ER pattern was defined as a J-point elevation of ≥0.1 mV above the baseline that was either notched (a positive J deflection at the QRS-complex/ST-segment transition) or slurred (a smooth transition from QRS to the ST segment) (Sarkozy et al., 2009).

**Table 1:** **Characteristics in Brugada syndrome patients**.

**Patient No**.	**Age years**	**Sex**	**History of VF**	**FH of SCD**	**Induced VF**	**SCN5A mutation**	**Spontaneous type 1 ECG**	**Leads displaying ER pattern**	**Both inferior and lateral ER pattern**
1	39	M	Yes	No	No	No	No	I, II, aVL, V4-6	Yes
2	30	M	Yes	No	No	No	No	II, III, aVF, V6	Yes
3	28	M	Yes	No	Yes	No	No	II, III, aVF, V6	Yes
4	34	M	Yes	No	Yes	No	Yes	No	No
5	41	M	Yes	No	Yes	No	No	No	No
6	52	M	Yes	No	Yes	No	Yes	No	No
7	61	M	Yes	No	No	No	Yes	No	No
8	33	M	Yes	Yes	Yes	No	Yes	No	No
9	61	M	Yes	No	Yes	No	Yes	III	No
10	50	M	Yes	No	Yes	No	Yes	II, III, aVF	No
11	48	M	Yes	No	Yes	No	Yes	II, III, aVF	No
12	39	M	Yes	Yes	No	Yes	Yes	No	No

*M, male; VF, ventricular fibrillation; FH of SCD, family history of sudden cardiac death; Induced VF, VF was induced with programmed electrical stimulation; ECG, electrocardiogram; ER, early repolarization*.

**Table 2:** **Characteristics in control subjects**.

	***N* = 17**
Male	13 (76)
Age, years	48 ± 19
**Primary illness**	
Supraventricular tachycardia	6 (35)
Idiopathic VF without ER nor BrS ECG	4 (24)
Syncope due to non-arrhythmic disease	3 (18)
Syncope due to coronary vasospasm	2 (12)
VF episode due to coronary vasospasm	1 (6)
Premature ventricular contraction	1 (6)
Both inferior and lateral ER pattern	6 (35)
Syncope due to non-arrhythmic disease	3 (18)
Syncope due to coronary vasospasm	2 (12)
Supraventricular tachycardia	1 (6)
Only inferior ER pattern	1 (6)
Only lateral ER pattern	0 (0)
Pilsicainide challenge	16 (94)

*Values are given as mean ± SD or number (%)*,

*BrS, Brugada syndrome*.

### Electrophysiological study

**Figure 1:**
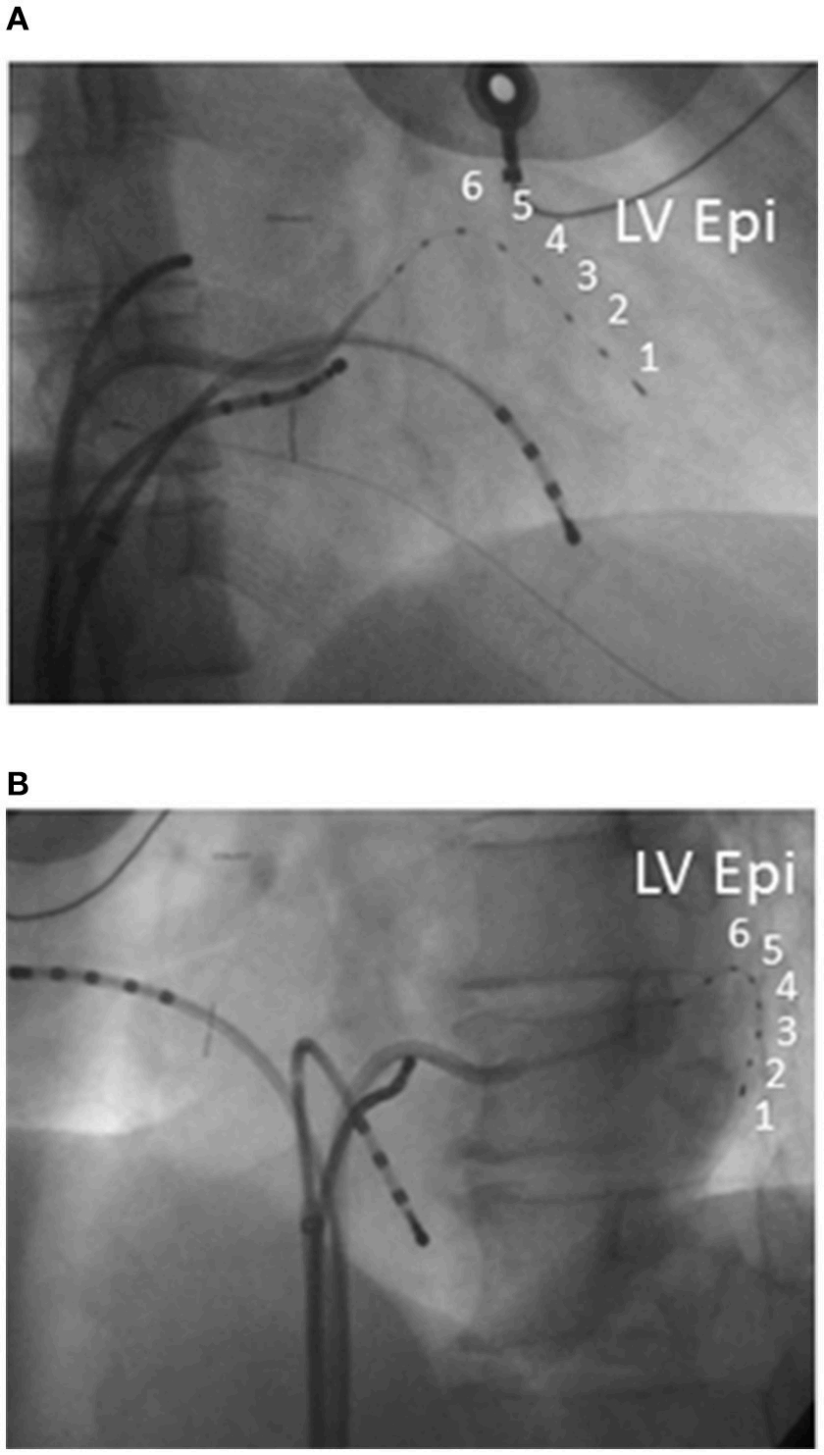
**Catheter position in electrophysiological study**. Right anterior **(A)** and left anterior **(B)** oblique view in electrophysiological study. The multipolar catheter was introduced into the left lateral coronary vein (LV Epi 1-6).

EPS was performed on all BrS patients and control subjects under local anesthesia. Written informed consent was obtained from all subjects before EPS. A maximum of three ventricular extrastimuli were delivered from the right ventricular (RV) apex and the RVOT unless VF had been induced at a previous step in all patients with BrS. To record the epicardial electrogram in the LV directly, we introduced a multipolar catheter (Ensemble FX1820, 8 pole; 5 mm interelectrode spacing; Japan Lifeline Co, Japan) into the lateral coronary vein on the left side retrogradely via the coronary sinus in all subjects (Figure 1). Angiography of the coronary vein or the coronary artery followed by coronary vein angiogram was performed in all subjects. Subjects who lacked lateral coronary veins were excluded. Accordingly, one BrS patient and two control subjects with supraventricular tachycardia were excluded before enrollment. We recorded local unipolar electrograms with a 0.05- to 100-Hz bandwidth and local bipolar electrograms with a 30- to 100-Hz bandwidth in the LV. The electrode of the catheter in the inferior vena cava was used as an indifferent electrode to record unipolar electrograms. Epicardial electrograms in the LV were recorded during sinus rhythm and constant right atrial pacing in all patients. Responsiveness to constant atrial pacing was evaluated at a maximum rate of 1:1 atrioventricular conduction. Epicardial J wave was defined as a distinct J-wave pattern or J-point elevation with a notch or slur at the terminal part of the QRS. The amplitudes of J waves were measured from baseline to peak of J wave and junction, respectively. The average value was calculated from the values measured in three consecutive beats during sinus rhythm. The maximum amplitude of each J wave recorded in the multipolar catheter was evaluated. Amplitude was compared between the last pacing beat and the first subsequent sinus beat. Duration of potential after the QRS complex in bipolar recording was also evaluated. Pilsicainide was administered at a dose of 1 mg/kg over a 5-min period in selected BrS patients. Isoproterenol was administered at a dose of 1 μg in one patient with BrS during LV epicardial mapping. A local endocardial electrogram was recorded with a quadripolar 6F deflectable catheter positioned at the endocardium in the LV in patients with both inferior and lateral ERs. This study was approved by the Ethics Committee of Okayama University.

### Genetic analysis

Genetic analysis was performed in compliance with the guidelines for human genome studies of the Ethics Committee of Okayama University in all BrS patients. Informed consent was obtained before analysis. The details of the DNA analysis have been reported previously (Nagase et al., 2008).

### Statistical analysis

Continuous variables were expressed as the mean ± SD. Means were compared between the two groups using Student's *t*-test or Mann-Whitney *U*-test. Differences between the two groups were analyzed using chi-squared test. *P* < 0.05 was considered statistically significant. JMP7 (SAS Institute, Cary, NC, USA) was used for analysis.

## Results

**Figure 2:**
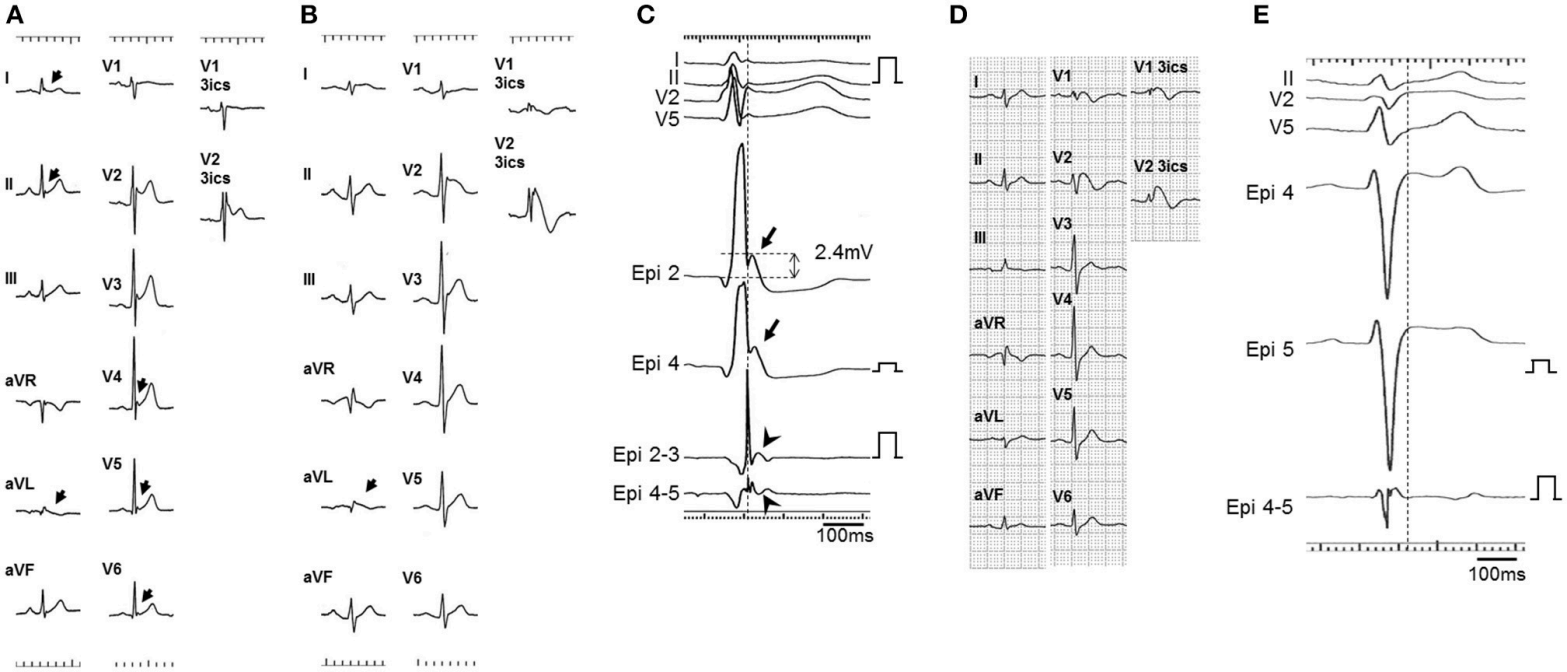
**ECG and epicardial electrograms in two BrS patients (patients 1 and 12)**. ECG at baseline **(A)** and after pilsicainide administration **(B)** in patient 1. ER pattern (arrows) was recorded in leads I, II, aVL, V4, V5, and V6 at baseline and in lead aVL after pilsicainide administration. Type 1 ECG appeared in lead V2 on the third intercostal space (3ics) after pilsicainide administration. **(C)** Unipolar (Epi 2 and 4) and bipolar (Epi 2-3 and 4-5) electrograms on the left ventricular (LV) epicardium in patient 1. Prominent J waves (arrows) were observed on unipolar recording and potentials (arrow heads) were observed after the QRS complex on bipolar recording in the left lateral coronary vein. The J wave with unipolar recording almost coincided with the potential after the QRS complex with bipolar recording and also coincided with the ER pattern on ECG. The amplitude of prominent J wave in lead Epi 2 is 2.4 mV. ECG **(D)** and epicardial electrograms **(E)** in patient 12. ER was not recorded on ECG. Prominent J waves were absent from the unipolar recording (Epi 4, 5) and delayed potentials were absent from the bipolar recording (Epi 4-5).

**Figure 3:**
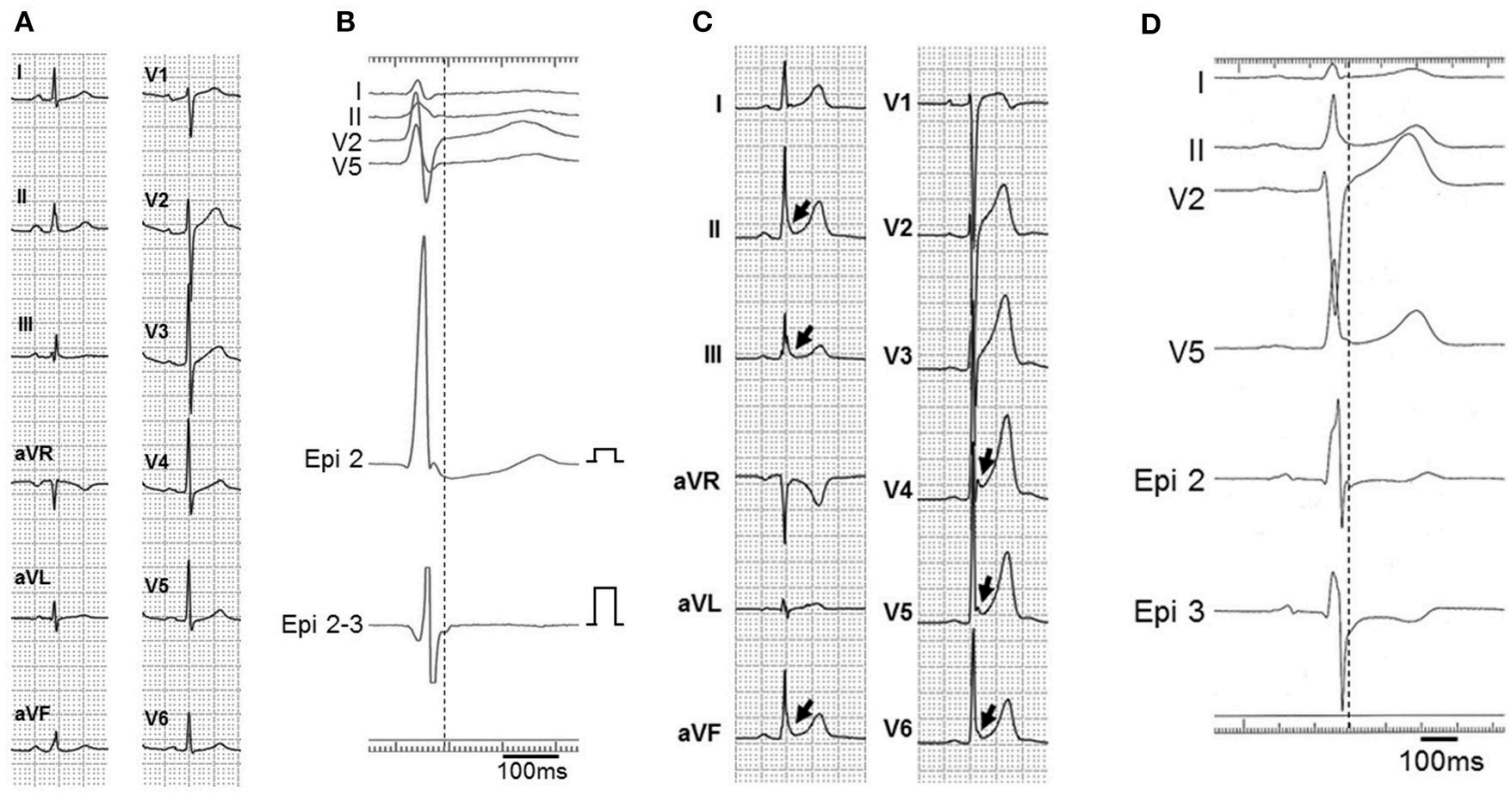
**ECG and epicardial electrograms in two control subjects. (A,B)** ECG without ER pattern and LV epicardial electrograms of a 39-year-old male control subject with supraventricular tachycardia. No apparent J wave was recorded on unipolar recording (Epi 2) and no potential was recorded after the QRS complex on bipolar recording (Epi 2-3) in the left lateral coronary vein. **(C,D)** ECG and LV epicardial electrograms of a 25-year-old male control subject with neutrally mediated syncope. ECG revealed inferior and lateral ERs (arrows). No apparent J wave was recorded on unipolar recording (Epi 2, 3).

**Figure 4:**
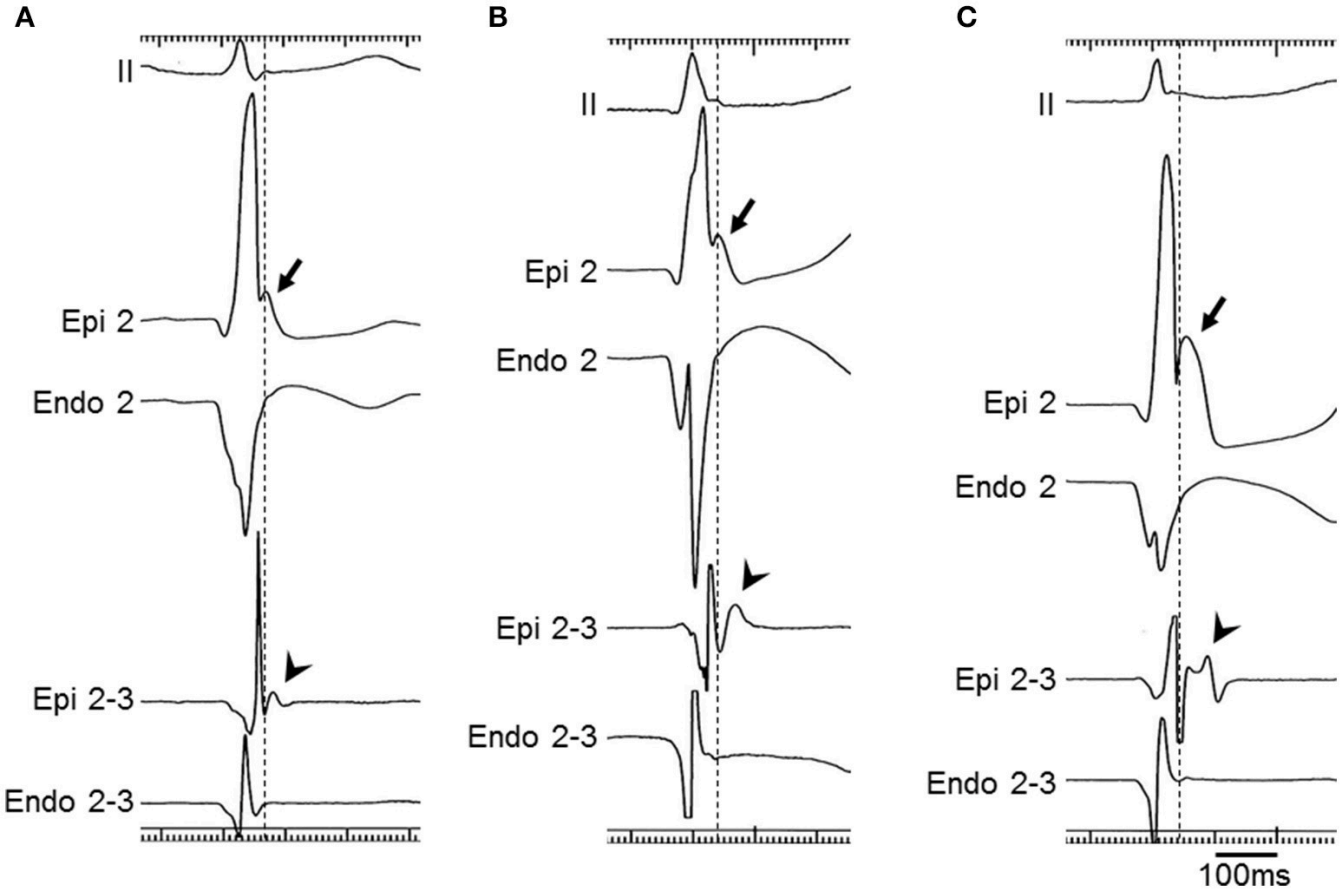
**Epicardial/endocardial electrograms in BrS patients (patients 1-3)**. ECG lead II and unipolar (Epi 2 and Endo 2) and bipolar (Epi 2-3 and Endo 2-3) electrograms on the LV epicardium (Epi 2 and Epi 2-3) and endocardium (Endo 2 and Endo 2-3) in patients 1, 2, and 3 **(A–C**, respectively). Prominent J waves were detected with unipolar recording (arrows) and potentials after the QRS complex were detected with bipolar recording (arrow heads) at the epicardium but not at the endocardium in all patients.

**Figure 5:**
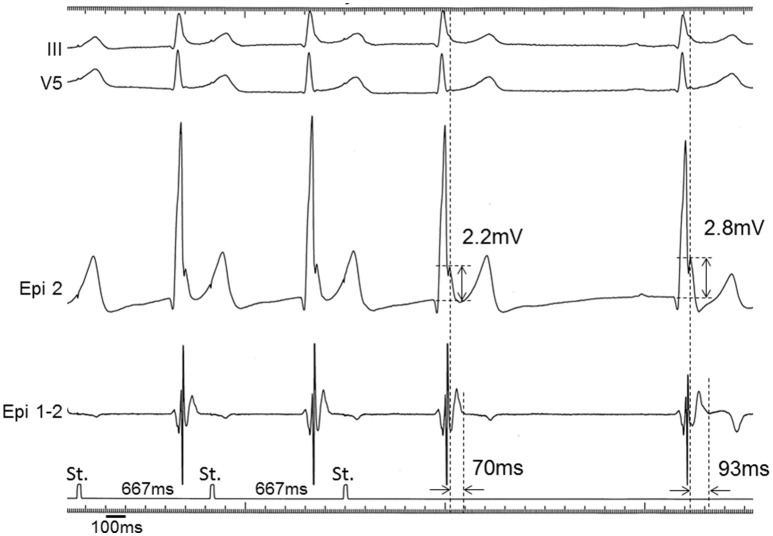
**Epicardial electrograms with atrial pacing in a BrS patient (patient 2)**. Unipolar (Epi 2) and bipolar (Epi 1-2) electrograms during and immediately after constant atrial pacing (cycle length = 667 ms) in patient 2. The J wave with unipolar recording was diminished (2.2 mV) and the duration of the potential after the QRS complex with bipolar recording was shortened (70 ms) during constant atrial pacing. Pause-dependent augmentations of the J wave amplitude and the duration of the potential after the QRS complex were observed (2.8 mV and 93 ms, respectively) following the first sinus beat.

**Figure 6:**
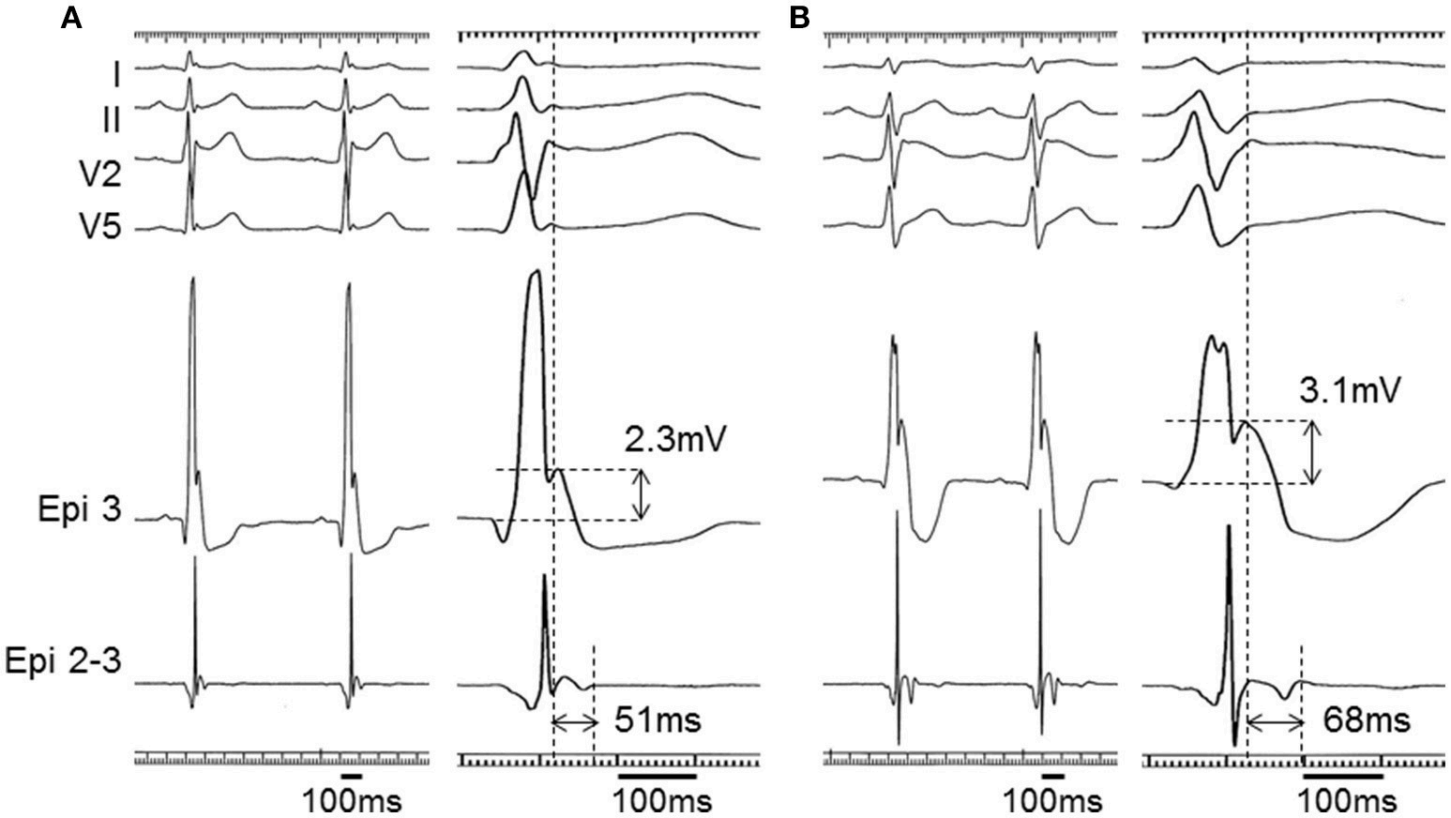
**Epicardial electrograms with pilsicainide administration in a BrS patient (patient 1)**. Unipolar (Epi 3) and bipolar (Epi 2-3) electrograms at baseline **(A)** and after pilsicainide administration **(B)** in patient 1. The J wave with unipolar recording was accentuated from 2.3 to 3.1 mV and the duration of the potential after the QRS complex with bipolar recording was prolonged from 51 to 68 ms by pilsicainide administration. The ER pattern was diminished, but the S wave also appeared in leads I, II, and V5 after pilsicainide administration. ECGs are also apparent in Figures 2A,B.

**Figure 7:**
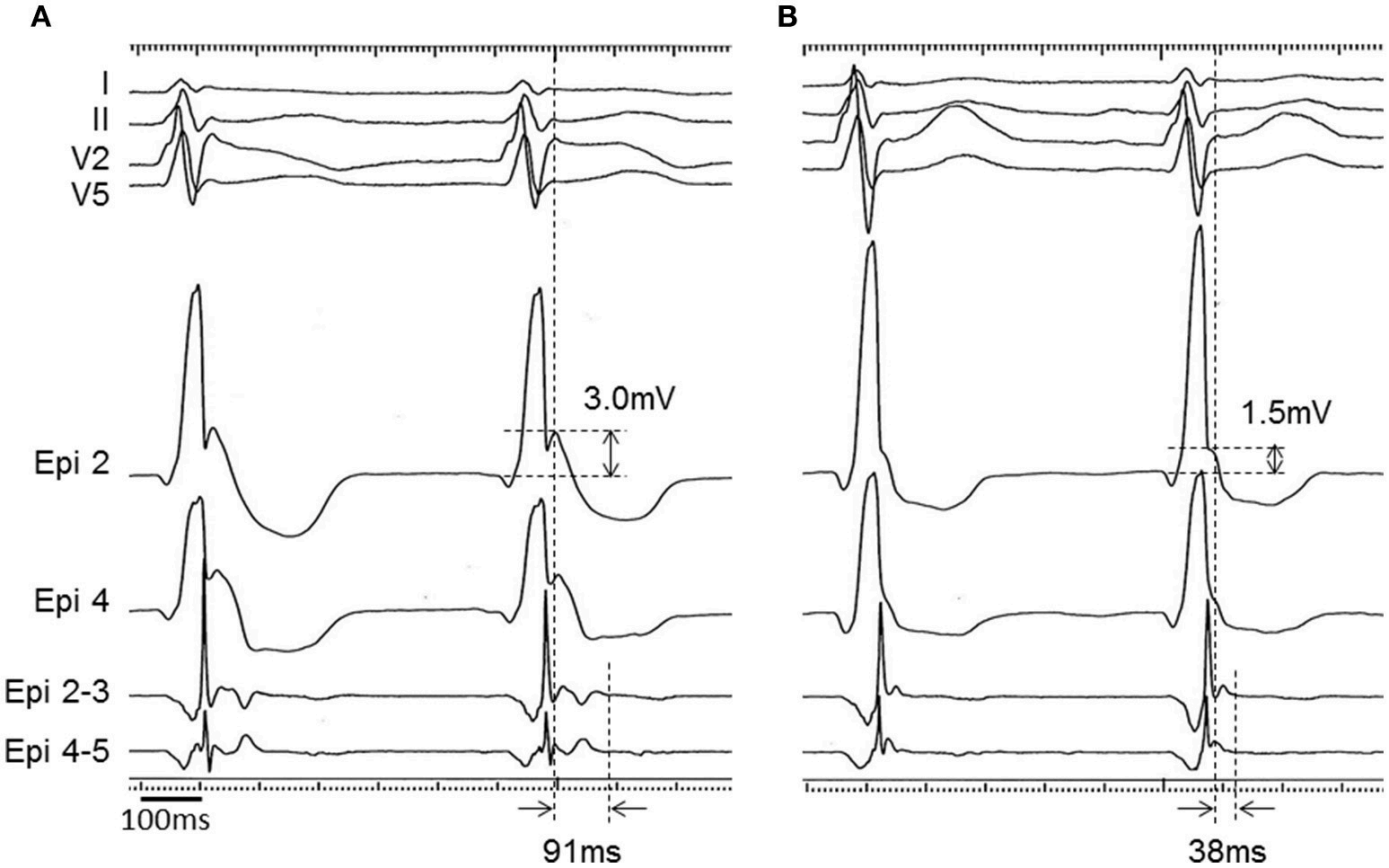
**Epicardial electrograms with isoproterenol administration in a BrS patient (patient 1)**. Unipolar (Epi 2 and Epi 4) and bipolar (Epi 2-3 and Epi 4-5) electrograms before **(A)** and after **(B)** isoproterenol administration in patient 1. J wave in the unipolar recording was diminished from 3.0 to 1.5 mV and potential after the QRS complex in the bipolar recording was shortened from 91 to 38 ms after isoproterenol administration.

Both inferior and lateral ERs were recorded in three patients with BrS (patients 1–3) and six control subjects. In the same three BrS patients, but not in any control subjects, prominent J waves of ≥2.0 mV were detected in unipolar recording at the epicardium and potentials after the QRS complex were detected in bipolar recording at the epicardium (Figures 2, 3). The J waves observed in unipolar recording coincided with the potentials after QRS complex observed in bipolar recording and also with the inferolateral ER patterns on ECG in patients 1–3. No J waves and no potentials after the QRS complex were recorded within the endocardium on the opposite side of the LV in any of these three patients (Figure 4). Pause-dependent augmentations of the J waves and potentials after the QRS complex followed by constant atrial pacing were observed in patients 1–3 (Figure 5). The J waves and potentials after the QRS complex on the LV epicardium were accentuated by pilsicainide administration in the two patients (patients 1 and 2) who received the pilsicainide challenge, though the inferolateral ER pattern was attenuated or disappeared (Figure 6). In patient 1, isoproterenol was administered after pilsicainide challenge, and the augmented J wave and potential after QRS were diminished (Figure 7).

## Discussion

The results of this study show that prominent J waves and potentials after the QRS complex can be recorded at the LV epicardium in BrS patients with global ER patterns. The prominent J waves and potentials after the QRS complex seen here were quite similar to those in our previously reported case of ERS (Nakagawa et al., 2014).

### Depolarization and repolarization abnormality

Antzelevitch and Yan proposed that BrS was one form of JWS, and that JWS was associated with prominent Ito mediated action potential notches in epicardial cells (Antzelevitch and Yan, 2010; Antzelevitch et al., 2016). Hoogendijk et al. reported that increased Ito can aggravate conduction across an isthmus and exaggerate ST-segment elevation in accordance with electrotonic current hypothesis based on activation block at sites of current-to-load mismatch (Hoogendijk et al., 2010, 2011; Ten Sande et al., 2015).

In BrS, several studies have reported the presence of depolarization abnormalities (Coronel et al., 2005; Wilde et al., 2010; Nademanee et al., 2015). Thus, depolarization abnormality on the RVOT epicardium is likely to prove an important mechanism for VF in BrS. Late potential (LP) on signal-averaged ECG (SAECG) has generally been recorded as abnormal depolarization. It is possible, however, that LP on SAECG may also represent a repolarization abnormality. Aizawa et al. reported that the LP on SAECG represents a repolarization abnormality in patients with BrS because the LP measured at 40 Hz with a low-cut filter disappeared when measured at 100 Hz in BrS, whereas the LP in patients with right ventricular cardiomyopathy was not affected by filter settings in this way, and the oral intake of quinidine, which confers a significant Ito blocking effect, eliminated LP in patients with BrS (Furushima et al., 2007; Aizawa et al., 2008; Hoogendijk et al., 2011). Abe et al. also reported that idiopathic VF patients with an inferior-lateral J wave had a high incidence of LP, showing circadian variation with night predominance (Abe et al., 2010). Exacerbations of the LP parameters were positively associated with the amplitude of the J wave.

### ER pattern and J wave with pilsicainide

Meijborg et al. reported that reduced sodium current in the left lateral ventricular myocardium cause inferolateral J waves on ECG and additional conduction slowing in the entire heart attenuated J waves because of masking by QRS prolongation (Meijborg et al., 2016). As in our previously reported case of ERS (Nakagawa et al., 2014), pilsicainide accentuated the LV epicardial J wave but diminished the ER pattern on ECG in both of the two patients with prominent J waves in the LV epicardium and ER patterns in the inferior and lateral ECG leads to whom it was administered (pilsicainide challenge was not administered to one patient with prominent J wave during EPS). This might be due to a transmural or far-field conduction delay causing the exaggerated J wave to merge with the S wave (Antzelevitch et al., 2016; Meijborg et al., 2016). This assumption is reasonable given that the right precordial lead is located near the RVOT epicardium, whereas the left precordial lead is far from the LV epicardium (Nagase et al., 2010; Brugada et al., 2015). This consideration is still hypothetical, however, and the results of this study should be confirmed in a larger number of BrS patients with inferolateral ER pattern as well as ERS patients.

### ER pattern and ventricular vulnerability

Several reports have suggested that the presence of an inferolateral ER pattern correlated with future arrhythmic events and electrical storms in BrS (Kamakura et al., 2009; Sarkozy et al., 2009; Kaneko et al., 2014). Our study showed that delayed potential coinciding with ER patterns on ECG can be observed in BrS patients on the LV epicardium as it has been on the RVOT. Pause-dependent augmentation of J waves is similar to that seen in patients with ERS (Aizawa et al., 2012). We suppose that a broader arrhythmogenic substrate would be associated with vulnerability to arrhythmia and that concomitant inferolateral ER patterns demonstrate the presence of an arrhythmogenic substrate on both RV and LV. Nevertheless, the precise mechanism linking ventricular vulnerability with ER patterns remains unresolved by our study.

### Epicardial unipolar recording

The significance of recording epicardial unipolar electrograms directly from the coronary vein has not been thoroughly investigated. Epicardial unipolar recording may enable us to analyze electrocardiographic and electrophysiologic properties, especially those concerning ER patterns/J waves and the ST segment. In some cases, local electrical phenomena are masked by far-field activity, so that ECG tends to underestimate the appearance of ER patterns/J waves and ST segments. Epicardial unipolar recording may yield more precise information concerning ER patterns/J waves and ST segments than ECG does. Epicardial mapping from the coronary vein allows us to acquire data only from the area through which the major epicardial vein runs. While the subxyphoid percutaneous approach to the epicardial space is particularly useful for precise epicardial mapping, this approach may not be feasible in BrS patients without symptoms and control subjects because of ethical concerns. Because coronary vein mapping is an invasive method that is sometimes abandoned due to the absence of suitable veins, this procedure may be of limited value for the prediction of future arrhythmic events. Epicardial mapping from the coronary vein can provide better stability during programmed pacing and/or drug administration, however, enabling us to avoid altering the ST segment through catheter contact.

### Epicardial electrogram on the RVOT and LV

In patients with BrS, epicardial electrogram recording revealed delayed potential on the RVOT and LV (Nagase et al., 2002). Furthermore, unipolar electrogram revealed J/ST-segment elevation followed by negative T wave, which was exaggerated by pilsicainide administration, on the RVOT and LV (Nagase et al., 2008). We could suppose that, although their ECG manifestations are different, epicardial delayed potential with prominent J wave is associated with type 1 ECG in the right precordial lead and ER pattern in the inferolateral ECG leads.

### Study limitations

The number of BrS patients, especially those with a global ER pattern, was small in this study. Accordingly, it is difficult to draw conclusions concerning the effects of programmed pacing and drug administration. This issue is a major limitation of this study. Because unipolar recordings are inevitably influenced by far-field electrical activity, a unipolar electrogram contains both local and far-field electrical information. Compared with ECG, however, epicardial unipolar recording may in fact yield more accurate information on local electrical conditions, especially with regard to J waves and ST segments. We were able to record electrograms only in the area through which the major epicardial vein in the LV runs. Epicardial recording was not performed on the anterior and inferior LV in this study. The number of control subjects was also small. Moreover, the characteristics of the control subjects were highly heterogeneous and were not matched with those of the BrS patients. Consequently, it was difficult to compare the two groups. LV endocardial mapping was not performed in the majority of patients because of ethical concerns. Endocardial mapping was performed only at the LV opposite the epicardial recording site in a small number of patients. Detailed endocardial and epicardial mapping acquired with an electro-anatomical mapping system should be evaluated in future studies. Programmed epicardial pacing on the LV was also not performed in this study.

## Conclusions

We recorded a prominent J wave in a unipolar electrogram and a potential after the QRS complex in a bipolar electrogram at the LV epicardium in BrS patients with inferior and lateral ER patterns on ECG. The prominent J waves coincided with the potentials after the QRS complex and with the inferolateral ER patterns on ECG. The characteristics of the inferolateral ER pattern on ECG in these patients primarily represent depolarization feature.

## Ethics statement

This study was carried out in accordance with the recommendations of Ethics Committee of Okayama University with written informed consent from all subjects. All subjects gave written informed consent in accordance with the Declaration of Helsinki. The protocol was approved by the Ethics Committee of Okayama University.

## Author contributions

SN: Design, data acquisition, data interpretation, writing. MT: Design, data acquisition, data analysis, preparation of figures. HM, KaN, HI, and TO: Data interpretation. KoN: Data acquisition, data interpretation, preparation of figures. TW and MM: Data acquisition. NN: Data acquisition, data analysis. KK: Data analysis, data interpretation, revising. All authors meet the following criteria: (1) Substantial contributions to the conception or design of the work; or the acquisition, analysis, or interpretation of data for the work, (2) Drafting the work or revising it critically for important intellectual content, (3) Final approval of the version to be published, (4) Agreement to be accountable for all aspects of the work in ensuring that questions related to the accuracy or integrity of any part of the work are appropriately investigated and resolved.

### Conflict of interest statement

The authors declare that the research was conducted in the absence of any commercial or financial relationships that could be construed as a potential conflict of interest.
